# Recurrent Parastomal Hernia Treated With Stoma Relocation and Abdominal Lipectomy: A Case Report

**DOI:** 10.7759/cureus.100347

**Published:** 2025-12-29

**Authors:** Rakan Mal, Khalid Alshehri, Raneem Alathath, Nouf Almalki, Mohamed Elkilani

**Affiliations:** 1 Department of General Surgery, King Abdulaziz Hospital, Jeddah, SAU; 2 Department of General Surgery, King Faisal Specialist Hospital and Research Centre, Jeddah, SAU; 3 Department of General Surgery, International Medical Center, Jeddah, SAU; 4 Faculty of Medicine, University of Jeddah, Jeddah, SAU

**Keywords:** abdominal wall reconstruction, hernia recurrence, onlay mesh, parastomal hernia, stoma relocation, sugarbaker technique

## Abstract

Parastomal hernia (PSH) remains a challenging complication following stoma formation, with recurrence rates remaining high despite advances in surgical techniques, including laparoscopic approaches. Recurrent PSH often necessitates complex reconstructive procedures to restore abdominal wall integrity.

We report the case of a 75-year-old female with a history of abdominoperineal resection and permanent end colostomy for rectal cancer who developed a PSH nine years after her initial surgery. She underwent a laparoscopic Sugarbaker repair but experienced recurrence within six months. A subsequent open abdominal wall reconstruction was performed, including lipectomy, stoma relocation, and onlay reinforcement with a biosynthetic mesh, as dense intra-abdominal adhesions precluded retromuscular mesh placement. The postoperative course was uneventful, and no clinical or radiologic evidence of recurrence was observed at short-term follow-up of six weeks.

This case highlights the technical challenges associated with laparoscopic repair in recurrent PSH and demonstrates that open reconstruction with stoma relocation and onlay mesh reinforcement may be a feasible option in selected complex cases. However, given the limited duration of follow-up, long-term durability and recurrence rates cannot be assessed.

## Introduction

Parastomal hernia (PSH) is the most common long-term complication following stoma formation, with reported incidence rates ranging from 4% to 56%, depending on stoma type, patient-related factors, and duration of follow-up [1,2]. It is estimated that approximately 50% of patients undergoing stoma formation will develop PSH after two or more years of follow-up [3]. End colostomies carry the highest risk, followed by loop colostomy and loop ileostomy [3]. Currently, only about one in three patients with PSH undergo surgical repair, which is partly attributable to the substantial recurrence rates observed after intervention [4]. Despite advances in operative techniques, recurrence following PSH repair remains a significant and unresolved clinical problem.

Common surgical approaches for PSH repair include stoma relocation, primary fascial repair with sutures, and mesh-based fascial reinforcement performed via open or laparoscopic techniques. Over the past decade, the laparoscopic Sugarbaker technique has gained widespread acceptance due to initially favorable outcomes compared with other methods, such as keyhole repair. However, despite these reported advantages, recurrence after Sugarbaker repair remains frequent, with published rates ranging from 8% to 56%, influenced by factors such as mesh fixation failure, mesh migration, and patient comorbidities [5,6]. Although technical modifications - including fascial defect closure and the use of newer mesh materials - have been proposed to reduce recurrence, systematic reviews indicate that no single technique has demonstrated consistent superiority [7,8].

In this case report, we present a 75-year-old female with a PSH at an end colostomy created nine years earlier following abdominoperineal resection. The patient initially underwent laparoscopic Sugarbaker repair but developed early recurrence within six months, necessitating stoma relocation and complex abdominal wall reconstruction (AWR). This case highlights the challenges associated with recurrent PSH following Sugarbaker repair and underscores the need for individualized surgical strategies in complex presentations.

## Case presentation

A 75-year-old female with a medical history significant for hypertension, dyslipidemia, hypothyroidism, obesity (elevated BMI), and multiple prior abdominal surgeries presented with recurrent PSH. She had previously undergone abdominoperineal resection for rectal cancer, followed by adjuvant radiochemotherapy, nine years prior. A permanent left end colostomy was created at that time. The patient also demonstrated marked abdominal wall laxity, which, together with obesity and prior operative interventions, represented significant patient-related risk factors for hernia development and recurrence.

**Figure 1:**
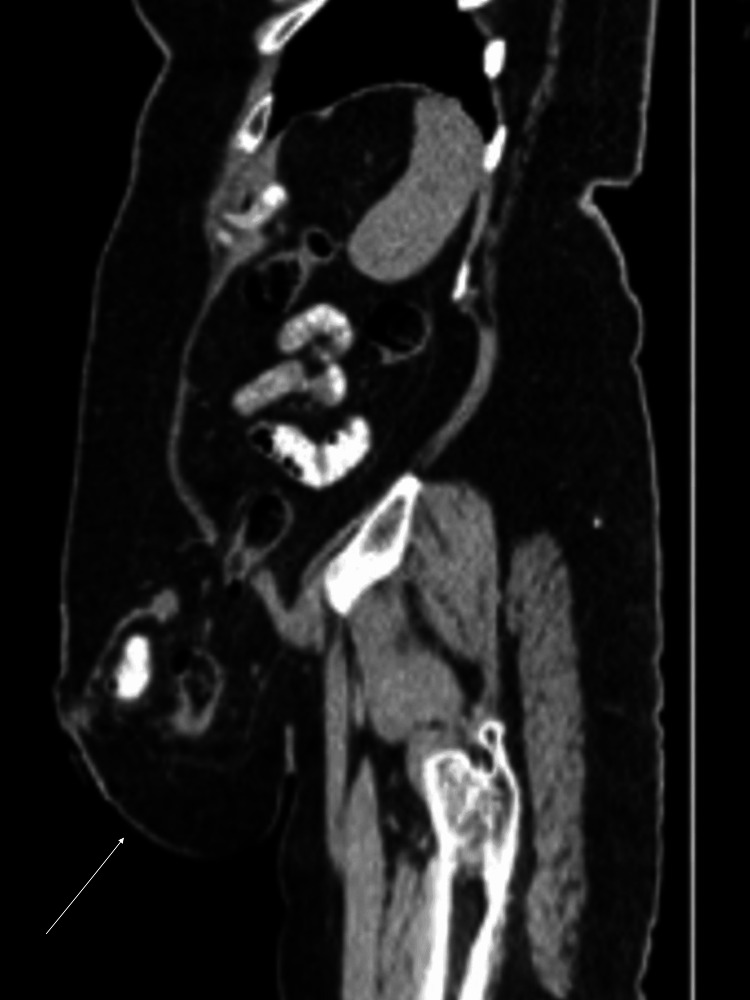
Initial CT scan at presentation showing parastomal hernia containing small bowel loops without obstruction.

She presented to our clinic with recurrent severe abdominal pain localized to the left lower quadrant parastomal region, persisting for four days. On initial evaluation, a computed tomography (CT) scan performed on December 25, 2022, demonstrated a left lower quadrant PSH containing small bowel loops without evidence of bowel obstruction. Additionally, two small infraumbilical and umbilical fat-containing hernias were identified, without associated complications (Figure 1).

Initial surgical management

To address the PSH, the patient underwent laparoscopic PSH repair on February 6, 2023, using the Sugarbaker technique. The procedure included adhesiolysis of the omentum and small bowel, followed by reduction of the hernia contents. The parastomal fascial defect was closed using a non-absorbable V-Loc™ suture (Medtronic/Covidien, Minneapolis, MN) to minimize tension around the stoma. A Symbotex™ Composite Mesh (Medtronic/Covidien, Minneapolis, MN), measuring 20 × 25 cm, was placed intraperitoneally to cover the stoma site and secured using a Capsure™ Fixation System (B. Braun, Melsungen, Germany) along with Prolene 2-0 sutures. The procedure was completed without intraoperative complications, and the patient remained hemodynamically stable in the immediate postoperative period.

**Figure 2:**
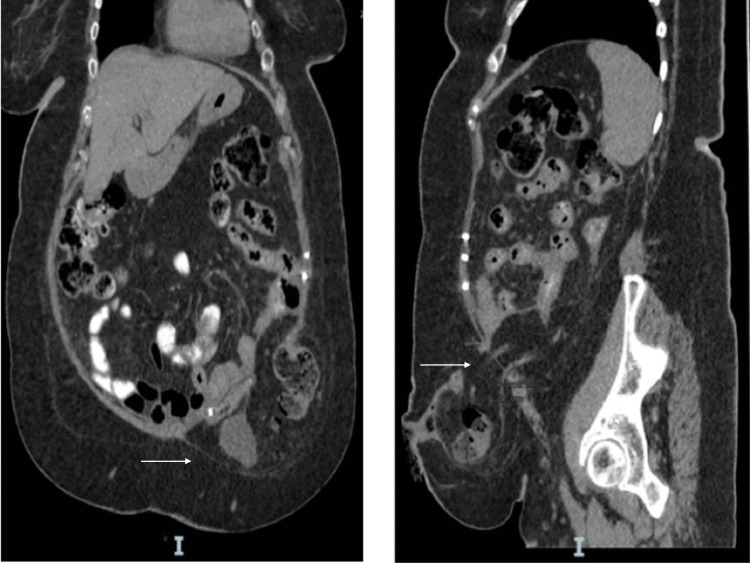
Follow-up CT abdomen and pelvis scan demonstrating recurrent parastomal hernia containing distal colon and omental fat six months post-initial laparoscopic Sugarbaker repair.

Despite the initial repair, the patient presented six months later with recurrent abdominal pain and tenderness at the stoma site. A follow-up CT scan performed on March 9, 2023, revealed a recurrent left iliac fossa PSH. The hernia sac measured 12.5 × 9.9 × 8.1 cm and contained distal colon and omental fat (Figure 2). Given the early recurrence following Sugarbaker repair and the patient’s predisposing risk factors, a decision was made to proceed with open repair and stoma relocation.

The patient was admitted electively and underwent open PSH repair with relocation of the stoma and onlay placement of the mesh on August 13, 2023. As a preparation, the patient received mechanical and chemical bowel preparation one day before the surgery. 

Surgical approach

The patient was transferred to the operating room and placed in the supine position. Following induction of general anesthesia and endotracheal intubation, a Foley catheter was inserted, and venous thromboembolism (VTE) prophylactic stockings were applied. Prophylactic antibiotics and anticoagulation were administered. The stoma appliance was removed under aseptic conditions.

**Figure 3:**
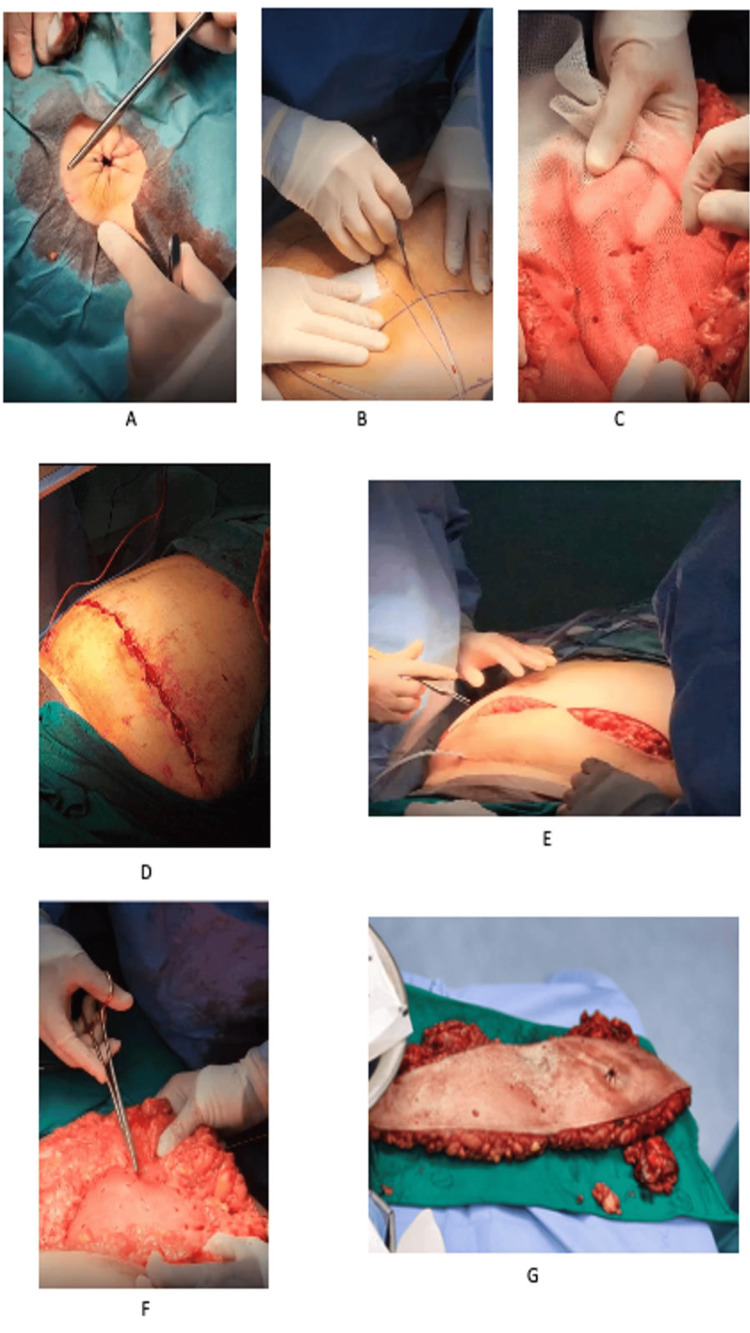
Intraoperative images showing onlay mesh placement and stoma relocation during open abdominal wall reconstruction (A-G). (A) Closing the skin defect of the previous stoma site. (B) A large elliptical incision to excise the excess skin along with previous stoma site. (C-D) Fixing a biosynthetic TIGR® Matrix mesh size 15 × 20 cm using AbsorbaTack™ and Vicryl™ 3-0 sutures. (E-F) Approximating the subcutaneous layer and closing the skin. (G) The excised excess skin along with the previous stoma site.

A circumferential incision was made around the existing stoma, and the colonic stump was transected using a gastrointestinal anastomosis (GIA) stapler. Hemostasis was achieved, and the skin defect was temporarily closed using a purse-string suture (Figure 3A).

A large elliptical incision was then created extending from the nipple line to the symphysis pubis to perform a lipectomy, reduce redundant skin, and facilitate stoma relocation (Figure 3B). The hernia sac was excised, and adhesiolysis was performed around the colon. A retromuscular plane was initially planned for mesh placement; however, dense intra-abdominal adhesions from multiple prior operations and loss of clearly identifiable tissue planes precluded safe posterior dissection. Given these intraoperative findings, the planned retromuscular approach was abandoned. The operative strategy was therefore modified to a tailored open repair incorporating stoma relocation, primary fascial closure, and onlay mesh reinforcement as a pragmatic and safer alternative in this specific clinical context.

Two additional lateral abdominal wall defects were identified and repaired using Vicryl 2-0 sutures. The colonic stump was refashioned, and the fascial defect at the original stoma site was closed in two layers. A new stoma was created through the previous muscular site with an adequately sized fascial opening. A biosynthetic TIGR® Matrix mesh (15 × 20 cm) was placed in an onlay position and secured using AbsorbaTack and Vicryl 3-0 sutures (Figures 3C-3D). The decision to use onlay biosynthetic mesh was guided by the patient’s history of multiple prior operations, risk of contamination, and the aim to reduce infectious complications.

The end colostomy was relocated to the left midclavicular line, approximately 5 cm superior to the incision. Two subcutaneous drains were placed bilaterally near the anterior superior iliac spine (ASIS). Local anesthesia with bupivacaine was administered, and the subcutaneous tissue was approximated with interrupted sutures (Figures 3E-3F). Skin closure was achieved using staples and Dermabond®, and the stoma was matured with Vicryl 3-0 sutures. Minor bleeding at the left drain site was controlled with compression. The patient tolerated the procedure well and was transferred to the intensive care unit in stable condition. An image of the excised skin and previous stoma site is shown (Figure 3G).

Postoperative follow-up

At her first outpatient follow-up on August 22, 2023, the patient was clinically well, with clean surgical wounds and no signs of infection. Surgical drains and skin staples were removed, and she was advised to continue mobilization and appropriate wound care.

**Figure 4:**
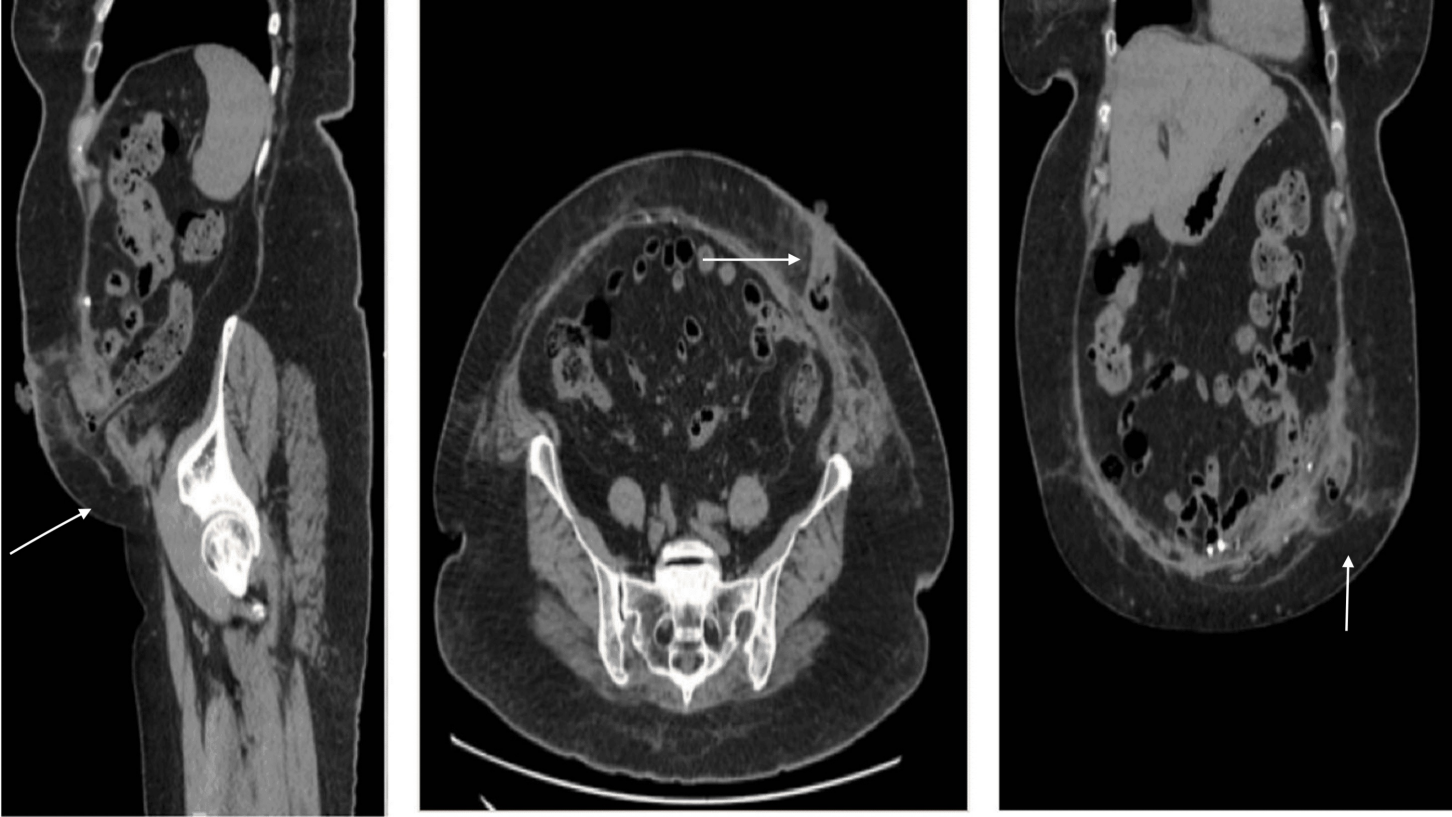
Postoperative CT scan confirming intact onlay mesh repair and absence of hernia recurrence at six weeks postoperatively.

A follow-up CT scan performed on September 26, 2023, confirmed appropriate mesh positioning with no evidence of recurrent herniation or postoperative complications (Figure 4). At the last documented follow-up, the patient remained stable, without recurrent abdominal pain or clinical signs of PSH recurrence.

Ethical considerations

Written informed consent was obtained from the patient for publication of this case report and the accompanying images.

## Discussion

Recurrent PSH remains a major challenge in abdominal wall surgery. In this case, the patient experienced early recurrence at six months following laparoscopic Sugarbaker repair, which necessitated subsequent open repair with stoma relocation and mesh reinforcement. This relatively early recurrence highlights the multifactorial nature of PSH failure, reflecting the combined influence of technical factors and patient-related risks rather than an isolated procedural deficiency. This case also underscores the importance of intraoperative adaptability, as the initially planned retromuscular strategy could not be safely completed due to operative findings.

The Sugarbaker technique, which involves intraperitoneal mesh placement with lateralization of the bowel, has been widely adopted due to reported lower recurrence rates compared with the keyhole technique [5]. Nevertheless, recurrence remains a significant clinical issue, with published rates ranging from 8% to 56%, depending on technical execution and patient characteristics [6,7]. Early failure after Sugarbaker repair may be related to inadequate mesh overlap, suboptimal fixation method or density, mesh migration, or insufficient bowel lateralization or orientation [9]. Current guidelines recommend a minimum mesh overlap of at least 5 cm to mitigate recurrence risk [10]. In addition, patient-related factors - including obesity, advanced age, abdominal wall laxity, and multiple comorbidities - have consistently been associated with higher failure rates [8]. In the present case, early recurrence likely reflected the cumulative effect of these patient-specific risk factors despite appropriate mesh sizing, in keeping with prior studies emphasizing the importance of careful risk stratification alongside meticulous surgical technique [7,8].

When PSH recurs, the decision between laparoscopic re-repair and open surgery must be individualized. Although minimally invasive approaches may reduce postoperative morbidity, they are often unsuitable in the presence of dense adhesions or large hernia defects. In our patient, the hernia measured 12.5 × 9.9 × 8.1 cm, and extensive adhesions limited safe laparoscopic access [7]. Open surgery provides superior exposure and allows stoma relocation and abdominal wall reinforcement when required. While complex open repairs can offer improved mechanical control, outcomes remain dependent on surgeon expertise and patient selection, with reported recurrence rates of 10-15% in specialized centers [11,12].

According to the European Hernia Society (EHS) classification system, the hernia can be specified as a Type IV PSH, given its recurrent nature, large size, and the presence of associated abdominal wall defects [12]. Use of the EHS classification improves standardization and allows clearer comparison with previously reported cases.

In the present case, dense intra-abdominal adhesions precluded access to the retromuscular plane, necessitating onlay mesh placement as a salvage reinforcement strategy rather than a component of formal AWR. This represents a modification of the initially intended operative plan based on intraoperative findings. Although onlay mesh is less commonly employed in PSH repair and is not considered part of standard AWR techniques, it remains a reasonable alternative when deeper anatomical planes are inaccessible. Evidence from incisional hernia repair suggests recurrence rates ranging from 5% to 20%, depending on technique and patient factors [13,14]. For PSH specifically, Amin et al. reported acceptable outcomes with onlay mesh placement and low associated morbidity [15]. While some meta-analyses report higher seroma rates with onlay techniques compared with sublay repairs, no consistent difference in recurrence has been demonstrated [13]. Importantly, the onlay position avoids intraperitoneal mesh contact and may reduce bowel-related complications such as erosion or adhesions [16].

A further consideration in this case was the use of a biosynthetic mesh. Biosynthetic materials, such as TIGR® Matrix, provide temporary mechanical support while gradually resorbing and allowing native tissue integration. This may be advantageous in complex or potentially contaminated fields and in patients with multiple prior operations, where permanent synthetic mesh may increase infection risk. Although long-term comparative data remain limited, biosynthetic meshes may represent a reasonable compromise between structural support and infection mitigation in selected complex cases.

Stoma relocation was undertaken due to compromise of the original site and inability to achieve a tension-free repair. We do not advocate stoma relocation as a routinely adopted strategy for recurrent PSH. Rather, it should be considered selectively when local tissue quality is poor or adequate repair cannot be achieved at the original site. Reported recurrence rates at the new stoma site range from 1% to 32% [12]. Previous studies suggest that retaining the stoma at the original site may predispose to persistent recurrence, whereas selective relocation may be associated with favorable outcomes in appropriately chosen patients [4,15].

A major limitation of this report is the short follow-up duration of six weeks, which is insufficient to assess long-term durability or true recurrence rates. The patient remains under planned long-term surveillance, and ongoing follow-up is required to evaluate late recurrence and mesh-related complications. Accordingly, the management described should be interpreted as our preferred approach in this specific case rather than a standardized or universally applicable strategy. Further studies with longer follow-up are required to better define optimal patient selection and long-term outcomes.

## Conclusions

This case illustrates a potential surgical strategy for the management of complex recurrent PSH, emphasizing the importance of individualized operative planning. While the Sugarbaker technique remains a widely accepted first-line approach, recurrent cases may require alternative open strategies when standard minimally invasive or retromuscular options are not technically feasible. In this patient, intraoperative findings necessitated abandonment of the initially planned retromuscular approach, prompting a tailored operative modification. In this patient, posterior component separation with transversus abdominis release (TAR), including a midline TAR approach toward the stoma site, was considered. However, dense intra-abdominal adhesions and loss of identifiable retromuscular planes at the stoma level precluded safe posterior dissection and mesh placement. Under these circumstances, a tailored open approach incorporating stoma relocation, primary fascial closure, and onlay mesh reinforcement was selected as a pragmatic solution.

Importantly, this report represents a single case with limited short-term follow-up, and, therefore, no conclusions regarding efficacy, superiority, or long-term durability can be drawn. The management described reflects our preferred approach in this specific clinical scenario and should not be interpreted as a standardized or universally applicable strategy. Rather, this case demonstrates one possible surgical option for selected patients with complex recurrent PSH when conventional techniques, including TAR-based reconstruction, are not achievable. Further comparative studies with longer follow-up are required to better define optimal patient selection and long-term outcomes for the various reconstructive strategies.
